# Thermal microclimate assessment in dairy cow milking parlors: Seasonal variations in temperature-humidity index and implications for heat stress

**DOI:** 10.14202/vetworld.2025.2024-2030

**Published:** 2025-07-22

**Authors:** Dimo Dimov, Toncho Penev, Ivaylo Marinov

**Affiliations:** 1 Department of Ecology and Animal Hygiene, Faculty of Agriculture, Trakia University, 6000, Bulgaria; 2 Department of Animal Husbandry, Ruminant Animals and Animal Products Technologies, Faculty of Agriculture, Trakia University, 6000, Bulgaria

**Keywords:** dairy cows, heat stress, microclimate, milking parlor, seasonal variation, temperature–humidity index, thermal comfort

## Abstract

**Background and Aim:**

Rising global temperatures and increasing humidity levels are intensifying the risk of heat stress (HS) in high-yielding dairy cattle. The temperature–humidity index (THI) is a standard metric for evaluating thermal stress in livestock. This study aimed to assess seasonal and diurnal variations in temperature, relative humidity, and THI within a milking parlor and determine their compliance with established thermal comfort thresholds for dairy cows.

**Materials and Methods:**

The study was conducted in a glass-roofed, windowless milking parlor housing 400 Holstein–Friesian cows in Bulgaria. Microclimatic parameters (temperature, relative humidity, and THI) were measured during three daily milking sessions (morning, noon, and evening) at 3 time points (start, middle, and end) over a 12-month period. Measurements were taken inside the parlor and 10 m outside. Statistical analysis involved one-way analysis of variance and *post hoc* tests using STATISTICA version 10.

**Results:**

Summer and spring exhibited the highest mean and peak temperatures (up to 31.4°C), while winter showed the highest relative humidity (82.39%). THI values peaked in summer, reaching levels classified as “danger” for dairy cows. Morning milking generally recorded lower temperatures and THI. Seasonal variation significantly influenced all microclimatic indicators (p < 0.001), while milking sequence significantly affected temperature and THI (p < 0.05).

**Conclusion:**

In-parlor thermal conditions, especially during summer, exceeded comfort thresholds and posed a risk for HS. The study underscores the urgent need to revise livestock housing regulations to include THI-specific standards for milking parlors. Incorporating real-time microclimatic monitoring can enhance animal welfare and productivity in dairy systems.

## INTRODUCTION

Global warming has continued its upward trajec-tory over recent decades, with tangible impacts now observable across various regions of the world [1]. Projections suggest that by 2100, the average glo-bal temperature could rise by 1.1°C to 6.4°C relative to 2010 levels [1, 2]. As one of the most pressing challenges of the 21^st^ century, climate change is poised to profoundly affect both ecological stability and agricultural productivity worldwide [3].

Among livestock species, dairy cows are parti-cularly vulnerable to elevated ambient temperatures and humidity, which hinder evaporative cooling – the primary mechanism for heat dissipation. When the ambient temperature becomes equal to or higher than that of the animals, they experience serious difficulties in cooling down and the risk of heat stress increases [4]. Heat stress (HS) in dairy cattle can be episodic or persist as a chronic condition, and it typically results from the combined effects of temperature and humidity, two critical environmental stressors [5].

To quantify the risk of HS in animal husbandry, the temperature–humidity index (THI) is widely employed as a practical and effective bioclimatic indicator [6]. In situations where solar radiation and wind data are unavailable, THI, derived from temperature and humidity, provides a reliable means of assessing ther-mal stress [7]. Numerous studies across diverse climates have highlighted the negative effects of HS on dairy performance. Of particular concern is the ability of high productive dairy breeds to adapt to increasingly harsh thermal environments [8, 9].

In the scientific literature, most of the studies on the effects of HS in dairy cattle, much of the existing research has focused on outdoor environmental conditions or barn-level climate control. Comparatively little attention has been paid to the microclimatic conditions within milking parlors – enclosed environments where cows spend some time during daily operations. This oversight is critical, as the thermal environment inside milking parlors can differ substantially from barn conditions due to architectural constraints (e.g., poor ventilation, heat-retaining materials, or direct solar exposure through glass structures located on the roof for better lighting). Moreover, most studies fail to account for variations across different times of day and seasons, which are known to influence the THI and thereby affect animal welfare and productivity. There is a lack of continuous real-time, high-resolution data capturing intra-day and seasonal fluctuations in microclimatic indicators during milking sessions. In addition, regulatory guidelines often generalize environmental thresholds without distinguishing between living and working spaces, such as milking parlors, thus potentially underestimating localized HS risks.

The present study aimed to evaluate the thermal microclimate within a commercial dairy cow milking parlor across all seasons and milking sessions of the day. Specifically, it sought to (1) monitor and quantify intra-parlor temperature, relative humidity, and THI during morning, noon, and evening milking; (2) assess temporal and seasonal patterns in microclimatic conditions using standardized measurements; and (3) determine whether recorded values align with the regulatory comfort thresholds established for dairy cattle welfare. By generating detailed environmental profiles of the milking parlor, this study also aims to provide evidence-based insights to support the development of microclimate-specific welfare standards and climate-resilient infrastructure designs for dairy production systems.

## MATERIALS AND METHODS

### Ethical approval

The study was conducted with the agreement of Protocol No 107 from the Ethics Committee of Trakia University, Bulgaria.

### Study period and location

This study was conducted from May 2018 to May 2019 at a commercial dairy farm located in a transitional continental climate zone in Bulgaria. The farm houses 400 Holstein–Friesian cows and features an eight-unit double-up “Herringbone” milking system. The milking parlor, in continuous use for 12 years without significant structural or ventilation upgrades, lacks windows and has a transparent glass roof that permits passive solar gain. The main barn is semi-open and fitted with a ther-mally insulated roof. Mechanical ventilation is limited to fans installed above the feed aisle.

### Milking routine and experimental design

Milking was carried out three times daily – morning, noon, and evening – with each session lasting approximately 2.5 h. Environmental monitoring was structured around these fixed time points to capture diurnal variability.

### Environmental data collection

Temperature, relative humidity, and THI were measured at three points during each milking session: The start, middle, and end. Measurements were collected monthly over a full 12-month period. Readings were taken at cow height in the center of the milking parlor to reflect actual exposure conditions. To enable comparative analysis, the same parameters were recorded simultaneously 10 m outside the milking facility.

### Instrumentation and calibration

**Figure 1:**
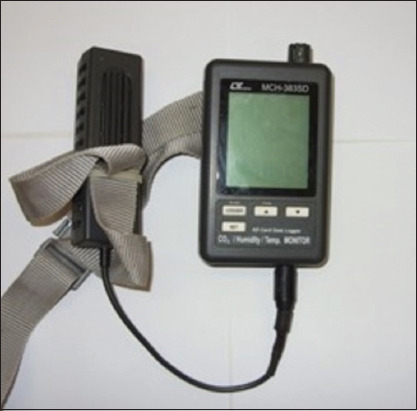
Lutron MCH-383SDB.

In-parlor temperature and humidity readings were obtained using a Lutron MCH-383SDB thermohygro-meter (Lutron Electronic Enterprise Co., LTD., Taiwan) (accuracy ±0.5°C for temperature and ±3% RH for hu- midity), calibrated according to manufacturer standards (Figure 1).

**Figure 2:**
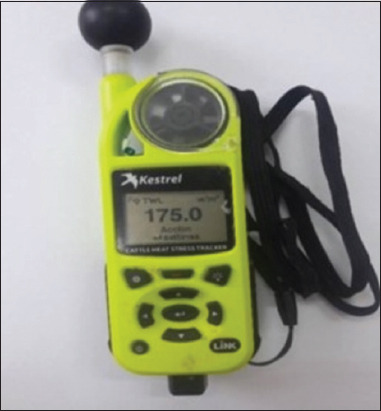
Weather meter Kestrel 5400 cattle heat stress tracker.

THI values were determined using a Kestrel 5400 Weather Meter (Kestrel Instruments, USA), which computes real-time HS indices based on ambient data (Figure 2). This high-resolution method enhances the precision of microclimatic assessments and is particularly useful for identifying thermal fluctuations that are often overlooked in broader environmental monitoring. All instruments were maintained and recalibrated according to factory specifications before data collection.

### Data processing and statistical analysis

Preliminary data entry and organization were performed in Microsoft Excel 2016 (Microsoft Corp. Washington, USA). Statistical analyses were carried out using StatSoft STATISTICA version 10 (TIBCO Software Inc., USA). Descriptive statistics (means and standard errors) were computed for all parameters. One-way analysis of variance (ANOVA) was conducted to evaluate the effects of season, milking sequence, and time within milking on the measured indicators. Significance was accepted at p < 0.05. Data homogeneity was assessed using Levene’s test, and *post hoc* comparisons were conducted using the least significant difference test.

## RESULTS AND DISCUSSION

### Climatic context of the study site

The study was conducted in a transitional cont-inental climate zone, typified by moderate winters and hot summers. Historical temperature records indicate average January temperatures ranging from −1.5°C to +1°C and July temperatures between 22°C and 24°C, with maximum summer values reaching 40°C [10]. Rec-ent meteorological trends show a clear rise in summer temperatures, corroborated by the findings of Stojnov *et al*. [11], who observed persistent periods of elevated temperature and THI in Southern Bulgaria. This farm was therefore selected due to its climatic predisposition for HS, especially during the summer months.

### Seasonal variation in temperature, humidity, and THI

Table 1 summarizes the seasonal mean and peak values for air temperature, relative humidity, and THI within the milking parlor.

**Table 1:** Average daily and maximum values of air temperature, relative humidity, and THI by season in the milking parlor.

Season	Number of observations (n)	Temperature (°C)		Humidity (%)		THI	
		
		X ± SE	Maximum	X ± SE	Maximum	X ± SE	Maximum
Summer	27	25.30 ± 0.43	31.4	62.60 ± 1.29	78.0	73.41 ± 0.55	80.0
Autumn	18	11.37 ± 0.53	14.4	67.46 ± 2.97	85.5	53.19 ± 0.88	57.92
Winter	9	12.90 ± 0.29	13.9	82.39 ± 2.32	89.8	55.53 ± 0.42	57.09
Spring	21	21.90 ± 0.95	31.4	62.51 ± 1.90	87.8	68.49 ± 1.29	78.06

THI=Temperature–humidity index, SE=Standard error

#### Temperature trends

The highest average and peak daytime tempe- ratures occurred in summer and spring, both reaching a maximum of 31.4°C. While spring’s average temper-ature was approximately 4°C lower than sum- mer’s, extreme peaks were comparable. Autumn and winter temperatures remained within moderate ranges of 11°C–14°C [12]. According to Perissinotto *et al*. [13], the thermoneutral zone for dairy cows ranges from 4°C to 26°C, with optimal performance expected within this range. Bulgarian legislation (Ordinance No. 44) recommends indoor cow housing temperatures bet- ween 10°C and 15°C, with an upper limit of 28°C [14]. In this study, in-parlor average temperatures frequently exceeded these thresholds, particularly in summer, suggesting a clear risk for the onset of HS.

#### Relative humidity patterns

The highest mean relative humidity was observed during winter (82.39%), approaching the maximum permissible limit of 85% set by Bulgarian standards. Across the other seasons, values ranged between 62.51% and 67.46%, remaining within acceptable thresholds. However, significant seasonal peaks were also noted – up to 89.8% in winter, exceeding comfort standards. This aligns with previous findings by MAFWE [14], Ozhan *et al*. [15], and Sagsoz *et al*. [16], which report frequent humidity violations in dairy facilities. While no specific humidity guidelines exist for milking parlors, the winter values approached levels that could adversely affect both animal welfare and equipment performance.

#### THI dynamics and HS risk

THI values were highest in summer, with averages exceeding 73 and peaking at 80, indicative of moderate HS. In spring, the mean THI was 68.49, with a maximum of 78.06, indicating a mild-to-moderate stress category. Based on published thresholds [17], THI values can be interpreted as follows:

- <68 = No stress
- 68–71.9 = Mild discomfort
- 72–74.9 = Discomfort
- 75–78.9 = Danger signal
- 79–83.9 = Danger
- ≥84 = Emergency.

Our results ranged from “mild discomfort” to the lower “danger” category. Previous research by Ishida *et al*. [18] indicates that THI values as low as 55 can reduce milk production, with other studies identifying upper tolerance limits of 66 for Japanese Holsteins [19].

### Effect of milking time and sequence on microclimate

#### ANOVA results for influencing factors

Table 2 presents the results of the analysis of variance for the influence of controlled factors on temperature, humidity, and THI.

**Table 2:** Analysis of variance for the influence of controlled factors on temperature, humidity, and THI.

Source of variation	Degrees of freedom (n – 1)	Temperature (°°C)		Humidity (%)		THI	
		
		MS	FP	MS	FP	MS	FP
Total number of models	7	403.79	31.27***	536.85	5.68***	827.92	32.68***
Season	3	887.90	68.78***	1047.4	11.08***	1854.5	73.21***
Milking sequence	2	71.21	5.52**	246.3	2.60-	99.2	3.91*
Reporting during milking	2	0.49	0.04-	54.5	0.58-	0.7	0.03-
Error	79	12.91		94.6		25.3	

Significant:

* p < 0.05;

** p < 0.01;

*** p < 0.001. THI=Temperature–humidity index, MS=Mean square, F=Value of the factor, P*=*level of significance; “- “ lack of significance

- Season had a highly significant effect on all three parameters (p < 0.001).
- Milking sequence significantly affected air temperature (p < 0.01) and THI (p < 0.05).
- Time within milking sessions (start, middle, and end) did not show statistically significant differences, likely due to the short duration (2.5 h) of each session.

#### Indoor versus outdoor microclimate comparison

To evaluate the insulating efficiency of the par-lor, initial temperature readings (taken before the animal’s presence) were compared with outdoor values. Across all seasons, the difference was minor (approximately 2°C), with summer inside temperatures slightly lower than outside and the reverse for other seasons. However, these differences were statistically insignificant, indicating that the milking parlor lacked effective thermal insulation.

### Diurnal microclimatic fluctuations

#### Air temperature by milking sequence

**Figure 3:**
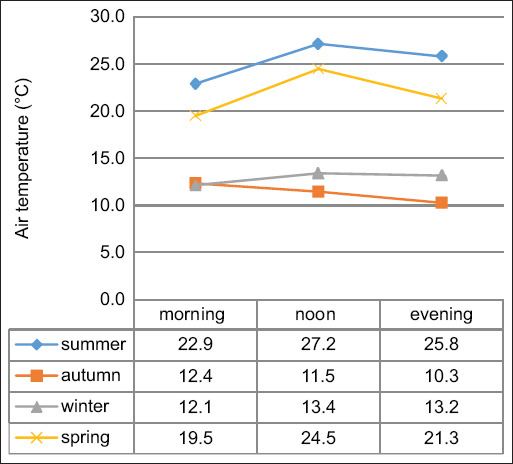
Least square mean values for air temperature in the milking parlor according to season and milking sequence.

Figure 3 shows Least Squares (LS) mean air temperatures by season and milking session. Morning milking consistently recorded the lowest temperatures, while noon sessions experienced peaks, except during autumn, when evening milking was coolest. The noon milking in summer posed the highest HS risk. As climate extremes become more frequent, it is essential to redesign dairy housing systems to maintain a stable internal environment [20].

#### Relative humidity by milking sequence

**Figure 4:**
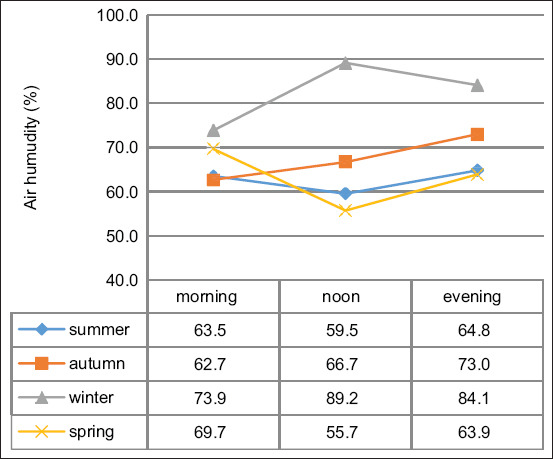
Least square mean values of the relative air humidity in the milking parlor according to season and milking sequence.

Figure 4 illustrates LS mean relative humidity values by season and milking sequence. In winter, the highest humidity was observed at noon. For other seasons, evening sessions showed elevated values, likely due to accumulated moisture from prior milking and cleaning. High humidity, particularly when combined with heat and solar gain, amplifies thermal discomfort [21].

#### THI variation by milking sequence

**Figure 5:**
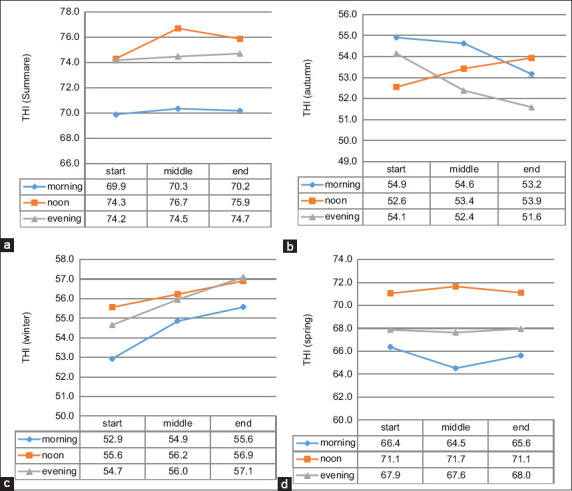
Least square mean temperature–humidity index (THI) values in the milking parlor during (a) summer, (b) autumn, (c) winter, and (d) spring.

As shown in Figure 5, THI values peaked during noon and evening milking in summer and during noon sessions in spring. Morning sessions consistently exhibited the lowest THI, apart from autumn. Findings by Penev *et al*. [22], Penev *et al*. [23], and Moallem *et al*. [24] support evidence that elevated THI reduces rumination and dry matter intake, ultimately decreasing milk yield. Timely monitoring of THI can aid in mitigating the negative impacts of HS, reinforcing its utility as a real-time farm management tool [25].

## CONCLUSION

This study revealed that the thermal environ-ment within the milking parlor of a commercial dairy farm significantly deviates from recommended comfort thresholds for dairy cows, especially during warmer seasons. The highest average and peak temperatures were recorded in summer and spring, reaching up to 31.4°C, while relative humidity peaked in winter (mean 82.39%), nearing the upper regulatory limit. The THI was consistently elevated in summer, with average values exceeding 73 and peaks reaching 80, classifying the microclimate as a moderate HS zone. Notably, season and milking sequence had a significant effect on thermal conditions (p < 0.001), but the milking parlor structure failed to buffer against external environmental variations, indicating poor insulation performance.

These findings underscore the urgent need for climate-adaptive designs in milking parlors. Modifying architectural elements such as roofing material, venti-lation systems, and insulation can help maintain thermal comfort and reduce physiological stress in dairy cows. In addition, scheduling adjustments, such as avoiding noon milking during summer or increasing cooling interventions during peak THI periods, may help mitigate HS effects on animal welfare and milk productivity.

A major strength of this work lies in its high-resolution, year-round monitoring of microclimatic variables during all daily milking sessions. By assessing temperature, humidity, and THI at multiple intra-day time points and across seasons, this study provides a granular temporal analysis often missing in similar research. The comparison between indoor and outdoor conditions further reinforces the validity of structural assessments related to thermal insulation.

While comprehensive, the study was confined to a single facility, limiting generalizability across different structural types or climatic regions. Furthermore, the lack of physiological and behavioral data on the cows (e.g., respiration rate, feed intake, and milk yield) prevents a direct linkage between microclimatic stressors and performance outcomes. Furthermore, solar radiation and air velocity were not recorded, which could provide a fuller picture of heat load dynamics.

Future research should include multisite evalua-tions across different barn configurations and clim-ates, integrate animal-level performance metrics, and test intervention strategies such as misting, shading, or structural retrofits. Moreover, the incorporation of automated sensors and internet of things-based monitoring can enhance real-time decision-making in climate-sensitive dairy management.

In summary, this study highlights a critical over- sight in dairy farm infrastructure – the thermal vulnerability of milking parlors. The current regulatory frameworks often neglect THI-based thresholds specific to these microenvironments. As global temperatures continue to rise, policy revisions and proactive design interventions will be essential to ensure the sustainability and welfare standards of modern dairy production. This research provides foundational data to guide such reforms and offers a scalable model for future assessments.

## DATA AVAILABILITY

The supplementary data can be made available from the corresponding author upon request.

## AUTHORS’ CONTRIBUTIONS

DD and TP: Conceptualization, methodology, data curation, formal analysis, data curation, and drafted the manuscript. IM, DD, and TP: Investigation, validation, and edited the manuscript. All authors have read and agreed to the published version of the manuscript.
